# Molecular and biochemical characterization of recombinant cel12B, cel8C, and peh28 overexpressed in *Escherichia coli* and their potential in biofuel production

**DOI:** 10.1186/s13068-017-0732-1

**Published:** 2017-02-27

**Authors:** Eman Ibrahim, Kim D. Jones, Keith E. Taylor, Ebtesam N. Hosseney, Patrick L. Mills, Jean M. Escudero

**Affiliations:** 1 Department of Environmental Engineering, Texas A&M University-Kingsville, Kingsville, TX 78363 USA; 2 Department of Botany and Microbiology, Al-Azhar University, Nasr City, Cairo, 11884 Egypt; 3 Department of Chemistry and Biochemistry, University of Windsor, Windsor, ON N9B 3P4 Canada; 4 Department of Chemical Engineering, Texas A&M University-Kingsville, Kingsville, TX 78363 USA; 5 Department of Basic Science, St. Louis College of Pharmacy, St. Louis, MO 63110-1088 USA

**Keywords:** Cellulases, Polygalacturonase, *Pectobacterium carotovorum*, *Escherichia coli*, Overexpression, Characterization, Biofuel, Catalysis, Optimization, Homology modeling, Crystal structure

## Abstract

**Background:**

The high crystallinity of cellulosic biomass myofibrils as well as the complexity of their intermolecular structure is a significant impediment for biofuel production. Cloning of *celB*-, *celC*-encoded cellulases (cel12B and cel8C) and *peh*-encoded polygalacturonase (peh28) from *Pectobacterium carotovorum* subsp. *carotovorum* (*Pcc*) was carried out in our previous study using *Escherichia coli* as a host vector. The current study partially characterizes the enzymes’ molecular structures as well as their catalytic performance on different substrates which can be used to improve their potential for lignocellulosic biomass conversion.

**Results:**

β-Jelly roll topology, (α/α)_6_ antiparallel helices and right-handed β-helices were the folds identified for cel12B, cel8C, and peh28, respectively, in their corresponding protein model structures. Purifications of 17.4-, 6.2-, and 6.0-fold, compared to crude extract, were achieved for cel12B and cel8C, and peh28, respectively, using specific membrane ultrafiltrations and size-exclusion chromatography. Avicel and carboxymethyl cellulose (CMC) were substrates for cel12B, whereas for cel8C catalytic activity was only shown on CMC. The enzymes displayed significant synergy on CMC but not on Avicel when tested for 3 h at 45 °C. No observed β-glucosidase activities were identified for cel8C and cel12B when tested on *p*-nitrophenyl-β-d-glucopyranoside. Activity stimulation of 130% was observed when a recombinant β-glucosidase from *Pcc* was added to cel8C and cel12B as tested for 3 h at 45 °C. Optimum temperature and pH of 45 °C and 5.4, respectively, were identified for all three enzymes using various substrates. Catalytic efficiencies (*k*_cat_/*K*_m_) were calculated for cel12B and cel8C on CMC as 0.141 and 2.45 ml/mg/s respectively, at 45 °C and pH 5.0 and for peh28 on polygalacturonic acid as 4.87 ml/mg/s, at 40 °C and pH 5.0. Glucose and cellobiose were the end-products identified for cel8C, cel12B, and β-glucosidase acting together on Avicel or CMC, while galacturonic acid and other minor co-products were identified for peh28 action on pectin.

**Conclusions:**

This study provides some insight into which parameters should be optimized when application of cel8C, cel12B, and peh28 to biomass conversion is the goal.

## Background

Production of biofuels from renewable resources has markedly increased in response to irregularities in the oil market and potential increases in fuel cost [1]. Second generation biofuel production from lignocellulosic biomass is an alternative strategy to mitigate greenhouse gas emissions and the high costs foreseen for first-generation biofuels derived from food crop resources [1]. Cellulose, a polymer of β-1,4-linked glucose, hemicellulose, a heteropolymer of d-xylose, l-arabinose, d-mannose, d-glucose, d-galactose, d-glucuronic acid, and lignin, a polymer of some phenolic components, in various proportions constitute the framework structure of plant biomass [2]. Enzymatic conversion of these polymeric compounds into various chains of fermentable sugars is one of the approaches for ethanol production [3]. However, the high crystallinity index of cellulosic microfibrils [3], and the complexity of the inter- and intramolecular hydrogen bonds and van der Waals interactions of the glucose residues [4], may counteract enzyme-surface accessibility and, hence, impede cellulose hydrolysis [5, 6]. Strain engineering, molecular analysis of native enzyme structures, protein synergies, and factors regulating enzyme biosynthesis have been outlined among several other factors in an attempt at improving the efficiency and cost of many lignocellulosic biomass conversion systems [7].

Designing a genetically modified bio-catalytic system with promise for lignocellulosic biomass conversions was carried out in the previous investigation [8]. Genes encoding cellulases, cel12B and cel8C, and polygalacturonase, peh28, of *Pectobacterium carotovorum* subsp. *carotovorum* (*Pcc*) have been selected in the previous investigation for their crucial role in plant cell wall maceration, as reported before [9, 10]. *Escherichia coli* (*E. coli*) was chosen as a convenient source of biocatalysts for biofuel production, due to its significant fermentation capacity with glucose [11], as well as several pentoses and other hexoses [12]. Genetic engineering of *E. coli* with the selected gene isolates of *Pcc* using a pTAC-MAT-2 expression vector and qualitative determination using CMC [13], for cel12B and cel8C, and polygalacturonic acid [14], for peh28, have been carried out [8]. The cloned genes were sequenced and their glycoside hydrolase (GH) families were identified with respect to the conserved domain sequences in the National Center for Biotechnology Information (NCBI) database [8]. Accordingly, GH families 12, 8, and 28 were the identified domain families for *celB*, *celC*, and *peh*, respectively. The conserved amino acid residues of the catalytically active sites were also assigned for each enzyme.

Cellulases of GH families operate through an acid–base catalytic mechanism with either inversion of configuration at the anomeric center, as in the GH-8 family, or retention, as in GH-12 family [15]. Cellulases with endolytic activity (endo-cellulases) generally have open active-site clefts that can bind to any region in the cellulose microfiber and hydrolyze the β-1,4-glycosidic linkages. On the other hand, the characteristic exo-cellulase active sites are tunnel-like to accommodate the end of a substrate [16], to produce oligosaccharides of different lengths [6]. Glutamic acid, Glu, and aspartic acid, Asp, are the conserved domain residues in the cel8C active site while two Glu, are the conserved domain residues in the cel12B active site [8]. The role of an Asp residue in the mechanistic pathway is to destabilize the internal sugar chain and direct the scissile glycosidic bond to the area of the proton donor [17]. The two Glu residues of the GH-12 family were found on opposite sides of the substrate-binding cleft and were proposed as the catalytic nucleophile and the Brønsted acid/base, effecting their catalytic actions in a double displacement, retention mechanism [18, 19]. Besides the catalytic domain of cellulases, other accessory domains such as carbohydrate-binding modules (CBMs) may also exist [20]. The role of a CBM in increasing the enzyme concentration on the polysaccharide surface [21], facilitating cellotetrose dissociation, [22], decreasing the biomass crystallinity index and increasing the yield of hydrolytic products [23], have been proposed [24].

On the other hand, the GH-28 family includes members with endo- and exo-polygalacturonase activities that may engage with other glycoside hydrolases in pectin disassembly [25]. Both endo-polygalacturonase (E.C.3.2.1.15) and exo-polygalacturonase (E.C.3.2.1.67) act by hydrolyzing the α-1,4-linked galacturonic acid residues of the homogalacturonan chains located in the smooth region of pectin. Endo-polygalacturonases hydrolyze the d-galacturonic acid residues within a set of homogalacturonan chains, while the non-reducing ends of galacturonan chains are the sites of activity in the case of exo-polygalacturonase [26]. Oligogalacturonates are the main products of the random hydrolysis pattern of endo-polygalacturonases (E.C. 3.2.1.15) on pectic acid, whereas monogalacturonate is the product of exo-polygalacturonase (E.C. 3.2.1.67) terminal action on the same substrate [27]. Asp active-site residues, Asp228, Asp249, and Asp250, were reported in our earlier investigation of the deduced amino acid sequence of peh28 [8]. These residues were found conserved among exo- and endo-acting polygalacturonases according to Abbott and Boratson [28]. Endo-polygalacturonases utilize the internal residues of the polymeric compounds by opening the surface cleft of the active site [29]. Loop insertion of certain amino acid residues was proposed to convert the active site from endo- into exo-activity by preventing enzyme accessibility to the internal residues of oligogalacturonates [28].

In this study, some of the molecular and mechanistic catalytic properties of cel12B, cel8C, and peh28 were investigated. The classification to Carbohydrate-Active enZymes (CAZymes) hydrolase families and the identification of the secondary and tertiary protein native structural features were facilitated using enzyme sequence homology modeling. The enzymes in their partially purified forms were characterized for their pH and temperature optima, substrate preferences, kinetic parameters, and product hydrolytic pattern. The synergy among cellulases was examined on crystalline and soluble cellulose derivatives using Avicel and carboxymethyl cellulose (CMC), respectively. Details for the structure and mechanism of action of the recombinant enzymes are provided in order to better characterize their industrial potential for biofuel production. This study is presented as a framework for our ongoing research on dynamic thermal characteristics as well as lignocellulosic biomass conversion using the tested enzymes.

## Methods

### Strains and media

Strains of *E. coli* DH5α (Lucigen, cat. no. 95040-456, Middleton, WI) harboring *celB*, *celC*, or *peh* plasmids; isolates of *Pectobacterium carotovorum* subsp. *carotovorum* (*Pcc*), ATCC™ no. 15359, [8], were used as sources of cel12B, cel8C, and peh28, respectively. Clones stored at −20 °C were cultured in Luria Bertani (LB) broth (cat. no. L3022), containing 100 µg/ml of ampicillin and incubated overnight at 37 °C with aeration. The freshly grown cultures of each respective clone were used for gene expression and enzyme extraction in the protocols discussed below. In this study, all chemicals were purchased from Sigma-Aldrich (St. Louis, MO) unless otherwise stated. Deionized water (DI H_2_O), nano-purified with a Barnstead Diamond™ Ultrapure water system (cat. no. D11901-7143, Thermo Scientific, Rockford, IL), was used throughout.

### Sequence analysis and homology modeling

The putative nucleotide sequences of cel12B, cel8C, and peh28 encoded genes have been analyzed and previously configured [8]. The nucleotide sequence similarities with other known published sequences were previously identified using BLAST-nucleotide (BLAST-n) of National Center for Biotechnology Information (NCBI) web-portal program (https://blast.ncbi.nlm.nih.gov/Blast.cgi) [8]. The isolated enzymes were designated for their respective glycoside hydrolase families using the BLAST server against NCBI-Conserved Domain Database (CDD), v 3.14 (NCBI-CD-BLAST) [8]. The molecular masses of the purified protein products of cel12B, cel8C, and peh28 were estimated using SDS-PAGE [8]. In this study, the homology of peh28′s deduced sequence to that of several pectate lyase and polygalacturonase I superfamily proteins of GH-28 was carried out using the NCBI-CD-BLAST program. Investigation of the carbohydrate-binding domain (CBD) with auxiliary and non-catalytic functions in both of the cel12B- and cel8C-deduced sequences was also carried out using the NCBI-CD-BLAST program. Protein–protein alignment-specific threshold value, bit score value, and the alignment significance expectation-value (*E*-value), were predicted for all of the tested protein residues with their corresponding aligned sequences using the NCBI reverse-position-specific-BLAST (RPS-BLAST) and the model’s position-specific scoring matrix (PSSM-47363), respectively. Theoretical isoelectric values and the potential *N*- and *O*-glycosylation sites in the deduced amino acid sequences were predicted using the JustBio-bioinformatics web-portal server (http://www.justbio.com/hosted-tools.html). Homology modeling was conducted using Phyre2-ProteinModel recognition, (v.2.0), web-portal server (http://www.sbg.bio.ic.ac.uk/phyre2/html/page.c-gi?id=index) [30]. The corresponding enzyme templates for cel12B, cel8C, and peh28 were selected based on their high relative identities to that of protein model native structure. The model proteins were tested for their alignment confidence with their template structures using Phyre2 server [28] in which both degrees of identities and the root mean square distance (rmsd) values of the aligned residues were assessed. The amino acid residues involved in the binding site clefts of the protein native structures were predicted using the web-portal 3DLigandSite recognition server (http://www.sbg.bio.ic.ac.uk/~3dligandsite/) [31], based on the similarity to other protein native structures. An open-source Java viewer for 3D-protein chemical structures (Jmol) (http://www.jmol.org/) was used for general analysis to the 3D-models generated. The high accuracy homology modeling of Phyre2 (core of the protein within 2–4 Å rmsd between the aligned set pairs) provides insight into the functional properties of the enzyme protein native structures.

## Biochemical characterization of recombinant cel12B, cel8C, and peh28

### Gene expression, enzyme extraction, and purification

Freshly inoculated *E. coli* strains harboring *cel*B, *cel*C, or *peh* were grown separately in LB broth containing 100 µg/ml ampicillin to an optical density of 0.5 at 595 nm. Gene expression was then induced by the addition of 0.1 mM isopropyl β-d-1-thiogalactopyranoside (IPTG) (≥99% (TLC), ≤0.1% dioxane, cat. no. 16458), and cells were harvested by centrifugation after 5 h, in the case of cel12B and cel8C, and 7 h, in the case of peh28. The empty vector strain was propagated and induced in the same manner as a negative control. Overexpressed soluble proteins were extracted and partially purified using the B-PER^**®**^ bacterial protein extraction kit (Thermo Scientific, cat. no. 90078, Rockford, IL) with DNAse (1, 2 and 500 U/ml), lysozyme (50 mg/ml), and a mild non-ionic detergent, such as Triton X-100, in 20 mM Tris–HCl buffer (pH 7.5). EDTA-free Halt Protease Inhibitor cocktail (Thermo Scientific, cat. no. 78425, Rockford, IL) was used at a final 1X conc. per ml of cell lysate mixture to prevent the possible proteolytic degradation during the process of cell lysis. Approximately 35 ml of the extract was then subjected to desalting and concentration using modified polyethersulfone, PES, ultrafiltration (UF) centrifugal techniques with different molecular weight cut-off (MWC), 50 mm PES membranes with 30 and 100 kDa MWCs (respective cat. no. MAP030C36 and MAP100C36, Pall Corporation, NY), and a 30 mm VIVASPIN^®^ 20 PES membrane with 50 kDa MWC (Sartorius, prod. no. VS2031, Thermo Scientific, MA) were used. In all cases, approximately 10 ml of the extracted protein solutions were introduced to the 100 kDa separating membrane, and centrifugation at 5000×*g* at 4 °C was performed for approximately 1 h. Sodium citrate buffer at 50 mM and pH 5.0 was used as exchange and washing buffer throughout the centrifugation period for both cellulase extracts, while sodium acetate at 50 mM and pH 5.0 was the exchange buffer used for polygalacturonase. The permeate fractions were then applied to the 50 and 30 kDa MWC membranes under similar conditions in order to concentrate further. The retentates of the 30 kDa separating device were collected in the cases of cel8C and PGase, however, the permeate fractions of the 30 kDa membrane treatment were collected in the case of cel12B. The extracted fractions were further purified by gel filtration chromatography using Sephadex G-100 (cat. no. G100120,) with a flow rate 0.75 ml/min in a CHROMAFLEX™ column of 120 cm length and 2.5 cm diameter (KONTES^®^, cat. no. 4208301210), using 50 mM sodium citrate buffer at pH 5.0 in case of the cellulases. Fifty mM sodium acetate at pH 5.0 was used as the elution buffer in polygalacturonase-containing fractions. A total of 60 fractions were collected and were tested for their cellulolytic or pectinolytic activities using 3,5-dinitrosalicylic acid (DNS) for cellulases, and copper and arsenomolybdate reagents, for polygalacturonase as described below. The fractions with the highest cellulase or polygalacturonase activities were selected for further characterization and purity determination. Sodium dodecyl sulfate-polyacrylamide gel electrophoresis, SDS-PAGE, was used previously for molecular mass identification of those eluted fractions with the highest activities [8]. The collected fractions were also analyzed for their protein content using a bicinchoninic acid (BCA) assay kit (cat. no. 23225, Thermo Scientific, Rockford, IL) with bovine serum albumin as the standard. The reduction of cupric ions, Cu^2+^ by the protein samples was detected using BCA working reagent, and the reaction was performed in an alkaline medium according to manufacturer’s instructions. The purple-colored solution thus generated was measured spectrophotometrically at 562 nm in reference to a blank of protein-free working reagent mixture.

### Polygalacturonase, cellulase, and β-glucosidase activity assays

Polygalacturonase activity of peh28 was measured based on a modified Nelson–Somogyi (NS) method [32, 33] with copper and arsenomolybdate reagents. The method is based on a redox reaction in an alkaline environment carried out by cupric ions of the Somogyi’s copper reagent on aldehyde groups in the hydrolysis products. The resulting reduced ion reacts further with the arsenomolybdate reagent forming a blue-colored product that can be detected at 520 nm within 0–250 µmol/ml product concentration range. In the typical assay, 0.2 ml enzyme solution was added to 0.5 ml of a 40 °C preheated mixture of 0.5% of polygalacturonic acid (sodium salt from citrus fruit, ≥75% titration, cat. no. P3850) dissolved in 50 mM sodium acetate buffer (pH 5.0). DI H_2_O was added to a final volume of 1.0 ml and the reaction was incubated for 1 h at 40 °C. The reaction was terminated by adding one volume of Somogyi’s copper reagent to each reaction, and the solution was boiled for 10 min in a dry bath.

After cooling to room temperature, one volume of Nelson’s arsenomolybdate reagent was carefully added with intermittent gentle mixing followed by the addition of 9.5 ml DI H_2_O and incubation for 10 min at room temperature for color stabilization purposes. The reaction mixture was centrifuged at 13,000 rpm for 1 min, and the change in the absorbance of the supernatants was detected at 520 nm using an Odyssey spectrophotometer model DR/2500 (cat. no. 5900000, Hach, Loveland, CO). A set of diluted standards was prepared and incubated in the same way using approximately 1 Unit/mg (U/mg) purified polygalacturonase from *Aspergillus niger* (E.C.3.2.1.15; cat. no. 17389). One unit of polygalacturonase activity is defined as the amount of enzyme releasing 1 µmol of reducing sugars per minute from polygalacturonic acid under the assay conditions.

Cellulase activities of cel12B and cel8C were determined using a modified 3,5-dinitrosalicylic acid (DNS) method of Miller [34]. The method is based on the oxidation of sugar aldehyde groups by DNS under alkaline condition with the formation of orange colored products which can be detected at 540 nm within the 100–500 μmol/ml concentration range. Both Avicel (50 μm particle size, cat. no. 11365) and the sodium salt of carboxymethyl cellulose (low viscosity, cat. no. C5678) were used as substrates for exoglucanase and endoglucanase activity determinations, respectively. Typically, crude/partially purified enzyme was added to a 45 °C preheated mixture of 2.0% substrate in 50 mM sodium citrate buffer (pH 5.0) and DI H_2_O was used to adjust the volume to 1 ml of reaction mixture. The reaction was incubated for 1 h at 45 °C and was terminated by adding 2.0 ml of DNS reagent and boiled 10 min in a 100 °C water bath. The samples were cooled to room temperature and the absorbance of the resulting products was measured at 540 nm. A 0.13 U/mg sample of cellobiohydrolase I (E.C.3.2.1.91) from *Hypocrea jecorina* (cat. no. E6412) and an approximately 1 U/mg purified product of 1,4-(1,3:1,4)-β-d-Glucan 4-glucanohydrolase (E.C.3.2.1.4) from *A. niger* (cat. no. 22178) were utilized for the calibration of standard curves for exoglucanase and endoglucanase activities, respectively, under similar assay conditions. One unit of cellulase activity is defined as the amount of enzyme releasing 1 µmol of reducing sugars per minute from CMC or Avicel under the assay conditions.

β-Glucosidase activities of cel12B and cel8C were determined using a modified method described by Parry et al. [35]. In this method, *p*-nitrophenyl-β-d-glucopyranoside (*p*NPG) (cat. no. N7006) was utilized as the substrate in a microtiter plate screening system (Benchmark microplate reader, cat. no. 170-6850, BioRad, Hercules, CA). The method was based on an indirect spectrometric quantification of the yellow colored product, *p*-nitrophenol. To initiate the reaction, the enzyme solution (crude or partially purified) was added to a 50 °C preheated mixture of 10 mM *p*NPG in 50 mM of sodium acetate buffer (pH 5.0) and DI H_2_O was used to make a final of 200 µl of reaction mixture. The reaction was incubated 30 min at 50 °C and was terminated by adding an equivalent amount of 0.4 M glycine–NaOH buffer (pH 10.8). The change of the absorbance at 405 nm was measured using the multi-well plate reader. A β-glucosidase calibrator equivalent to 0.25 U/ml (cat. no. KA1611, Abnova, Walnut, CA) was utilized to prepare a set of diluted standards. One unit of β-glucosidase activity is defined as the amount of enzyme releasing 1 µmol of *p*-nitrophenol per minute from *p*NPG under the assay conditions.

A triplicate set of reactions was set up for each enzyme measurement and substrate and enzyme colorimetric blanks were prepared following the enzyme assay protocol with water replacing that of substrate/enzyme complex. Possible enzyme interference with some other proteins of *E. coli* metabolism was excluded by including a control of lysates from an empty vector control strain. In all assay experiments, cel12B, cel8C, and peh28 were initially added at approximate concentrations of 0.8, 0.3, and 0.2 U/ml, respectively, based on a previous assessment of the enzyme optimum level producing activity.

### Mode of activity and substrate specificity of cel12B and cel8C

The substrate specificity of cel12B and cel8C and their modes of action as endoglucanase, exoglucanase, and/or β-glucosidase were investigated using 20 mg/ml CMC, 20 mg/ml Avicel, and 10 mM pNPG substrates, respectively. The enzymes were incubated individually with each substrate and were assayed using the corresponding assay method described above for cellulases and β-glucosidase.

### Reaction rate and catalytic rate constants of recombinant cel12B, cel8C, and peh28 on their respective substrates

Values of the Michaelis–Menten constant (*K*_m_), maximum enzyme velocity (*V*_max_), turnover number (*k*_cat_), and the specificity constant (*k*_cat_/*K*_m_) were assessed by measuring the enzyme initial activities over defined concentration ranges of their substrates. CMC at 1.0–40 mg/ml was used for cel12B or cel8C, while 0.05–0.55 mg/ml polygalacturonic acid was used for peh28. Enzyme initial activities were determined using the same experimental and assay conditions described above for each enzyme.

Fitting the initial activity and substrate concentration data to the Michaelis–Menten Eq. (1) was performed using GraphPad Prism v.5.1 (GraphPad Software Inc., La Jolla, CA), where $S$ is the substrate concentration (in mg/ml), $V_{\hbox{max} }$ is the enzymatic reaction rate (in µmol/ml/min) in which the enzyme active site is saturated by the substrate, and $K_{\text{m}}$ is the substrate concentration necessary for an enzyme to attain half of its maximum reaction rate. The data were utilized in calculating the enzyme turnover number (*k*_cat_), *V*_max_/*E*_T_, and the enzyme specificity constant (*k*_cat_/*K*_m_) on each substrate. *E*_T_ is the enzyme’s molar concentration in mM of a kinetic run which can be obtained by dividing the concentration of total protein in mg per ml of reaction solution by the enzyme’s molecular weight in mg per mmol.

1 $$v = \left( {V_{\text{max} } \left[ S \right]} \right)/\left( {K_{\text{m}} + \left[ S \right]} \right)$$

### Determination of pH and temperature optima for maximum substrate conversions with the recombinant enzymes

Optimum pH for cel12B, cel8C, and peh28 activities was investigated using 25 mg/ml of CMC for cel12B and cel8C and 4.0 mg/ml of polygalacturonic acid for peh28. The assays were performed following a similar protocol described above for cellulase and polygalacturonase activities except that a broader pH range, 3–10, was used herein. For pH adjustments, 50 mM sodium citrate buffer (pH 3.0–6.2), 50 mM Tris–HCl buffer (pH 7.0–9.0), and 50 mM glycine–NaOH buffer (pH 9.0–10.0) were used for cel12B and cel8C, while 50 mM sodium citrate buffer (pH 3.0–3.4), 50 mM sodium acetate buffer (pH 3.6–5.6), 50 mM citrate phosphate buffer (pH 5.8–7.0) along with the defined range of Tris–HCl and glycine–NaOH buffers were used for peh28.

Temperature optima for cel12B, cel8C, and peh28 activities were determined by setting up the assay experiments at various temperatures in the range 15–80 °C. The enzyme assays were performed at pH 5.4 in 50 mM sodium citrate and 50 mM sodium acetate for cellulase and polygalacturonase activities, respectively, using the same substrates and substrate concentrations described above for optimum pH investigation.

### Synergism of cel12B and cel8C on Avicel and CMC

Cel12B and cel8C were tested for their synergistic actions on CMC and Avicel by comparing their individual and combined activities on each substrate. The reaction mixture consisted of 50 mM sodium citrate buffer (pH 5.4), 5 mM MgSO_4_, 25 mg/ml CMC or Avicel, 0.334 U/ml of cel12B, and/or 0.816 U/ml cel8C. To rule out possible product inhibition, synergism of the tested cellulases with 0.5 U/ml of recombinant β-glucosidase was also examined using similar experimental conditions. β-glucosidase is a *Pcc*-*Bgl* pTAC-MAT recombinant clone overexpressed in *E. coli* and partially purified by PES membrane ultrafiltration and size-exclusion chromatography using Sephadex G-100 (Ibrahim et al., unpublished data). The reactions were allowed to proceed for 3 h at 45 °C with samples taken every 10 min in the first hour and every 20 min in the following hours for product quantifications. Measurement of the enzymatic hydrolysis products was carried out using the DNS assay method described above.

### Product analysis

Mono- and di-saccharide hydrolysis products were monitored over 3 h of combined activities of cel12B, cel8C and β-glucosidase on Avicel or CMC, using gas chromatography coupled with mass spectrometry (GC–MS). The reaction mixture consisted of 50 mM sodium citrate buffer (pH 5.4), 25 mg/ml CMC, or Avicel, 2.70, 1.81 and 0.5 U/ml cel12B, cel8C, and β-glucosidase, respectively. Hydrolysis by peh28 on pectin from citrus peel extract (cat no, P9135) was also monitored over 4 h using GC–MS. Pectin stock solution was made by dissolving 2% (w/w) pectin (Pectin from citrus peel, Galacturonic acid ≥74.0% (dried basis), Cat no. 9000-69-5) in DI H_2_O with 1N NaOH added to bring the pH to 5.4. The solution was then incubated at 45 °C for as long as 16–18 h and the microbial growth was inhibited by including tetracycline, cycloheximide, and chloramphenicol (cat. no. 87128, C104450, and C0378, respectively) at a final concentration of 0.1 mg/ml for each antibiotic. Peh28 was then added at 1.78 U/ml to a pectin solution (final concentration of 4 mg/ml) to initiate the reaction. All reactions were incubated at 45 °C and pH 5.4 and the hydrolysate aliquots were collected every 30 min and quenched with four volumes of ethanol (99.9%, HPLC grade, cat. no. V002075). The resulting suspensions were centrifuged for 5 min at 13,000 rpm at room temperature, and the reducing sugar products were analyzed in the supernatants as follows. Samples in appropriate quantities were dried in 1.5 ml amber glass GC vials (Supelco, cat. no. 27084-U, Bellefonte, PA) with thermoseal liners (Supelco, cat. no. 27191, Bellefonte, PA), under a stream of nitrogen in a concentration system (Barvap 12, Glas-Col, LLC, cat. no. 109A 11-12000, Terre Haute, IN) at 60 °C for 30–60 min. A blank containing the same set of reaction constituents was prepared for each enzyme and was terminated at time zero (*t* = 0) using ethanol.

Derivatization of hydroxyl groups of reducing sugar products via their *N*,*O*-bis[Trimethylsilyl]trifluoroacetamide (BSTFA) derivatives in the presence of other catalysts such as pyridine and trimethylchlorosilane (TMCS) has been previously reported [36]. Oxime derivative formation using hydroxylamine has been suggested as a precursor step to that of TMS-derivative formation to avoid sugar tautomer formation by the cyclic anomers of the latter compounds [37]. Parameter optimization for maximum oxime and TMS-derivative formation was carried out by Rivas et al. (unpublished data) based on the method of Willis [38]. For oxime formation, 300 µl of pyridine solvent (99.8%, anhydrous, cat. no. 270970), 300 µl of hydroxylamine hydrochloride (50 mg/ml in pyridine, cat. no. 159417), and 100 µl of salicin internal standard (2 mg/ml in pyridine, cat. no. S0625) were added to the dried sugars formed in the preceding steps. The reaction was incubated in a dry bath at 85 °C for 30 min and cooled to room temperature before proceeding to the next step. For TMS-derivative formation, 300 µl of BSTFA +1% TMCS (CAS#25561-30-2 (BSTFA) and CAS#75-77-4 (TMCS), Regis Technologies Inc., Morton Grove, IL) was added to the previous reaction mixture to make a total volume of 1.0 ml. The solutions were mixed thoroughly and were incubated for 30 min at 90 °C and for another 10 min at room temperature before analysis by GC–MS. The GC–MS analysis was carried out using gas chromatography (GC) (model 6890) coupled to a mass selective detector (MSD) (5973) and auto injector with a split/splitless capillary inlet system (model 7683) (Agilent Technologies, Inc. Hewlett-Packard, Santa Clara, CA). A 5% diphenyl, 95% dimethylpolysiloxane (HP-5MS) non-polar column (30 m length, 0.25 mm inner diameter, 0.25 µm film thickness, cat. no. 19091S-433, Agilent Technologies, Inc, J&W Scientific, Santa Clara, CA) was used for chromatographic separation of the derivatized compounds. Helium, at an inlet pressure of 14.9 psi and constant flow rate of 1.0 ml/min, was used as a carrier gas. The oven temperature was programmed to proceed from 180 to 300 °C at 15 °C/min over the course of 15 min. The MSD was operated in Electron Multiplier Voltage (EMV) mode at 1400 EM, mass range of 50–550 *m*/*z* at interface, and source temperatures of 150 and 230 °C, respectively. The injector was operated in a split mode with a split ratio of 1:10, injection port temperature of 250 °C, and injection volume of 1 µl. Data acquisition was done using MSD ChemStation software (E.02.01.1177, Agilent Technologies, Inc. Hewlett-Packard). The total ion current (TIC) chromatogram of GC elution and selective fragment ion (SFI) spectra of MS were used for sugar-identification where the SFI intensity and TIC retention time were used throughout for each eluted fragments as separated by GC–MS. Standard sugar oximes and/or TMS derivatives at 0-2.0 mg/ml concentrations of anhydrous d-(+)-glucose, d-(+)-galactose, d-(+)-mannose, d-(+)-xylose, l-rhamnose, d-(−)-fructose, sucrose, d-(+)-cellobiose, and d-(+)-galacturonic acid (cat. no. G8270, G0750, M6020, X1500, R3875, F0127, S0389, C7252, and 48280, respectively) were formed by the same procedure and used for peak identification. The mass spectrum of each standard was verified with the corresponding mass-spectrometry data of the National Institute of Standards and Technology (NIST) (http://webbook.nist.gov/cgi/cbook.cgi).

### Statistical analysis

Data in triplicate sets were analyzed using GraphPad Prism 6.0 (GraphPad software Inc., La Jolla, CA) and were compared using one-way analysis of variance (ANOVA) and Tukey post-test analysis as offered by the program.

## Results and discussion

### Sequence analysis and homology modeling

**Fig. 1:**
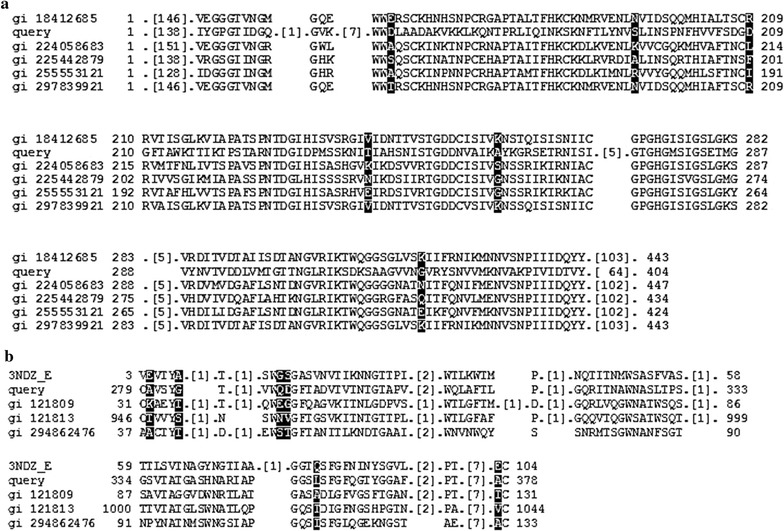
**a** Alignment of peh28-deduced residues with those of pectate lyase-6, cl19188, superfamily member PLN02793 as carried out using NCBI-CD-BLAST web-portal server. The Accession Numbers, gi 18412685, gi 224058683, gi 225442879, gi 255553121, and gi 297839921 represent the putative or hypothetical protein sources of *Arabidopsis thaliana*, *Populus trichocarpa*, *Vitis vinifera*, *Vitis vinifera*, *Ricinus communis* [58], while the tested peh28 sequence represented the *line query*. The *black-shaded area* represents the non-aligned sequences among all represented species compared to the others shown in the non-shaded region of the display. Peh28 showed high confidence similarity with the aligned protein sequences with overall domain specific threshold, bit score, of 116.13 and expectation-value, *E* value, 4.70e−29 from RPS-BLAST and PSSM mode of NCBI-CD-BLAST server, respectively. **b** Carbohydrate-binding domain (CBD) type II in cel112B protein sequence based on the alignment with those of other species using NCBI-CD-BLAST web-portal server. It shows two tryptophan residues that were found conserved among CBD-II of Pcc cel12B, query line, and the published sequences of endoglucanase D from *Clostridium cellulovorans*, 3NDZ_E, endoglucanase CelA from *Streptomyces lividans*, gi 121809, Cel12B from *Cellulomonas fimi*, gi 121813, and xylanohydrolase B from *Cellvibrio japonicas*, gi 294862476 according to Marchler-Bauer et al. [58]. The *black-shaded area* represents the non-aligned sequences among all represented species compared to the others shown in the non-shaded region of the display. Cel12B showed high confidence similarity with the aligned protein sequences with overall domain-specific threshold value, bit score, of 110.21 and *E* value, 1.40e-29 from the NCBI RPS-BLAST and PSSM mode servers, respectively

Successful cloning of full length DNA of *celB*, *celC,* and *peh* into *E. coli*, encoding for cel12B, cel8C, and peh28, respectively, has been previously reported [8]. The clones were confirmed for their sequence identities to that of *Pcc* polygalacturonase and cellulases as reported in accession numbers (Acc. No.) AAC02965.2, ZP_03832232.1 and AAA03624.1 of NCBI’S database [8]. Cel12B, cel8C, and peh28 were assigned to glycoside hydrolase (GH) families 12, 8, and 28, respectively, based on the homologies to the correlated domain sequences as described [8]. The work here is extended to further investigate the domain sequence similarities with others related in the NCBI database and to give some insight into the enzyme molecular structures based on the protein sequences of cel12B, cel8C, and peh28. Sequence homology of peh28 with endo-polygalacturonase (peh-1) and pectate lyase (pel-3) from *Pcc*, Acc. No. gb|L32172 was 99% based on NCBI-BLASTP analysis [39, 40] (figure not shown). However, peh28 was assigned to pectate lyase family 6, Acc. No. cl19188, based on the homology to that of conserved domain sequences of related proteins from other sources (Fig. 1a). Investigation of a carbohydrate-binding domain (CBD) in the cel12B and cel8C sequences was carried out using NCBI-CD-BLAST server. A CBD site of class II family with two conserved tryptophan (T) residues was found in the cel12B sequence as shown in Fig. 1b. The implication of conserved tryptophan residues in binding to crystalline cellulose has been previously investigated [41, 42]. No CBD site was, however, found in the cel8C sequence based on the same analysis.

Enzyme molecular weights of 29.5, 40, and 41.5 kDa were previously determined using SDS-PAGE for cel12B, cel8C, and peh28, respectively, [8], identical to the predicted values of each corresponding enzyme using the JustBio server (Table 1). Theoretical isoelectric points were also calculated for each protein sequence which were considerably higher for cel12B, 9.17, and peh28, 9.46, than that of cel8C, 7.73 (Table 1). This might indicate the presence of more positive residues on the protein surfaces of cel12B and peh28, relative to cel8C. Similar pI values were previously reported with other polygalacturonases and cellulases from different sources such as pIs of 8.73 and 8.45 for polygalacturonases NfPG I and NfPG III, respectively, from *Neosartorya fischeri* [43], pI 9.18 for endo-polygalacturonase-I from *Achaetomium* sp. [44] and pI 7.4 for an endoglucanase from *Trichoderma harzianum* (*T. harzianum*) [45]. Understanding the electrostatic interactions of the enzyme-ligand surface charges could have implications for enzyme productive binding in optimum biomass conversions [46].

**Table 1:** Isoelectric point (pI), molecular weight, and *N*-glycosylation sites predicted for cel12B, cel8C, and peh28

Enzyme	pI value	Molecular weight (kDa)^a^	*N*-Glycosylation sites
Cel12B	9.17	29.5	Asn^226^, Asn^230^, Asn^235^, Asn^280^, Asn^287^, Asn^308^, Asn^333^. Asn^393^, Asn^440^
Peh28	9.46	41.5	Asn^128^, Asn^161^, Asn^167^, Asn^207^, Asn^256^
Cel8C	7.73	40	Asn^128^

^a^The predicted molecular weights are in agreement with those identified for the enzymes using SDS-PAGE [8]

Several *N*-glycosylation sites were similarly predicted for the cel12B and peh28 sequences, unlike cel8C where only one site was detected (Table 1). *N*-glycosylation at the loop regions and/or near aromatic amino acid residues was found to provide structural stability to enzymes as discussed by Price et al. [47] and Culyba et al. [48]. The role of glycosylation in the cellulose-binding affinity of cellobiohydrolase has been previously reported [49]. No significant alteration in endoglucanase activity was detected, however, the enzyme hypo-glycosylation and hyper-glycosylation were carried out by expressing into *E. coli* and *S. cerevisiae*, respectively [50, 51].

**Fig. 2:**
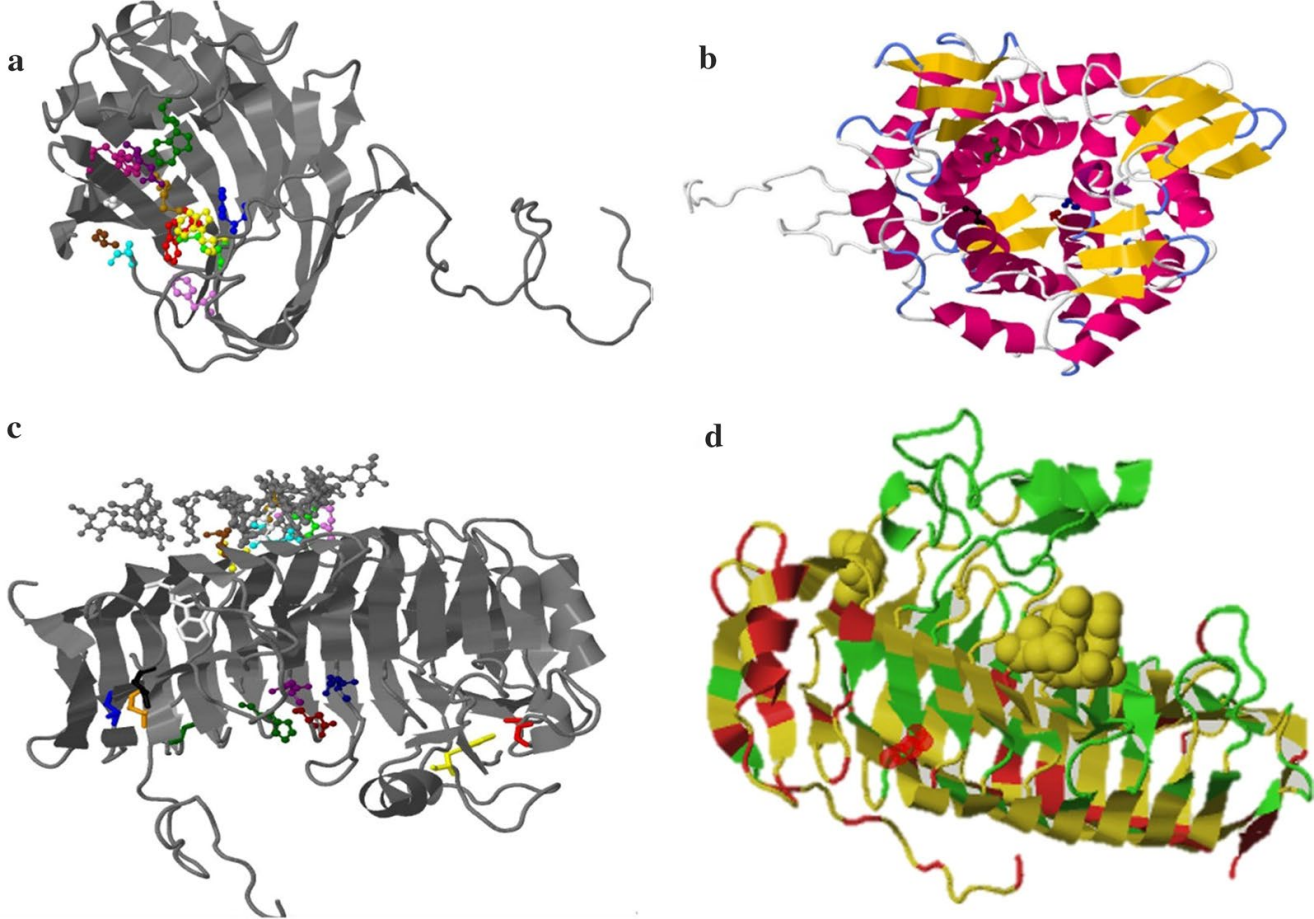
Schematic view of the protein 3D-model structures of **a** cel12B with β-jelly roll topology shown in the *gray*-ramped illustration. The predicted acid/base catalytic residues, Glu^158^ and Glu^246^, are shown in *purple* and *fuchsia ball* and *stick* representations in distinguishing from that of *yellow*, *blue*, *green*, *gold*, *red*, *aqua*, *brown*, *white*, and *violet* representations for Trp^56^, Tyr^92^, Trp^142^, Met^160^, Trp^162^, Pro^170^, Ala^171^, Ile^192^, Trp^200^, and Phe^248^ catalytic residues, respectively. **b** cel8C with α-barrel-fold architecture of six (α/α) helices structure shown as inner and outer layers of pinkish cartoon representations. The predicted catalytic residues, Glu^55^ and Asp^243^, are shown in the groove center in *green* and *blue ball* and *stick* representations in distinguishing from that of *red* and *black* representations for Tyr^244^ and Phe^335^ catalytic residues, respectively. **c** peh28 with right-handed β-helical-fold of ten full turns showing the *gray*-ramped cartoon representation. The predicted catalytic residues, Asp^228^, Asp^249^, Asp^250^, and His277, are shown in the* upper area* within the T-loop region with *blue*, *red*, *purple*, and *green ball* and *stick* representations in distinguishing from that are shown in the* bottom-sided area* with *yellow*, *aqua*, *gold*, *violet*, *lime*, *white*, and *brown* representations for Ser^27^, Asp^28^, Ser^29^, Arg^30^, Asn^237^, Asn^265^, Asn^290^ catalytic residues, respectively. Cys^115^ and Trp^160^ residues at the peripheral loop region, and Asn^370^, Val^367^, Val^368^, Trp^351^, and Val^330^ at the C-terminus are seen as *red*, *yellow*, *black*, *blue*, *orange*, *white*, and *green stick* representations, respectively. **d** Peh28 showing the alignment confidence values with endo-polygalacturonase I protein template of van Pouderoyen et al. [54]. High alignment values are shown in *red* and *yellow* representations, while the *green* displayed areas are of moderate alignment values according to Phyre2-model alignment investigation, [30]. The space-filling representations shown in the side and the center of the T-loop region are of Arg^96+^ and the catalytic residues of peh28, respectively. Those residues are at a high degree of alignment with those of polygalacturonase I as indicated by the *yellow-colored* representation shown in their displayed areas

Figure 2a–c show the protein model structures of cel12B, cel8C, and peh28, respectively, as predicted by Phyre2-protein model recognition server [30]. Accordingly, β-jelly roll topology was the fold architecture for the cel12B structure which showed 68% homology and 1.78 Å rmsd [30], with endo-β-1,4-glucanase chain B sequence from *Bacillus licheniformis* [52]. Cel8C, however, showed an α-barrel fold architecture with a pair of parallel six-helix domains located at opposite alignments and forming inner and outer rings in the model structure (Fig. 2b). The structure showed 58% structural identity and 2.20 Å rmsd [30], with that of Mazur and Zimmer for a related GH-8 endoglucanase sequence, BcsZ, from modified *E. coli* [53]. On the other hand, a fold of single-stranded right-handed β-helices with 10 full turns was identified for the predicted peh28 structure as shown in Fig. 2c. The similarity to that of endo-polygalacturonase I sequence from *A. niger* [54], was determined for the peh28-deduced sequence, with overall 95% homology and 1.90 Å rmsd [30]. Figure 2d shows the alignment confidence of *Pcc*’s peh28 and *A. niger*’s endo-polygalacturonase I sequences based on Phyre2-model-alignment assessment of Kelley et al. [30]. As illustrated, peh28 has high alignment confidence with the conserved domain residues of endo-polygalacturonase I as well as with other non-conserved residues such as those of the Arg^96^ residue. Arg^96^ has been suggested to guide the processive behavior of *A. niger*’s endo-polygalacturonase I through a flexible binding due to the substrate negative surface alignment with the enzyme active site [54]. The authors, van Pouderoyen et al., reported that mutagenesis of Arg^96^ to Ser yielded a non-processive mutant of endo-polygalacturonase I, thereby establishing the function. The high alignment of such a residue with that of *Pcc*’s peh28 suggests the processivity function of the latter enzyme. Moreover, the high homology of peh28 to the conserved domain sequences of *A. niger*’s endo-polygalacturonase I and that of pectate lyase family 6 would suggest the multi-domain characteristic and, hence, the dual functional properties of peh28. The tight linkages reported of pectate lyase, *pel*-*3*, and polygalacturonase, *peh*-*I*, genes in the *Pcc* chromosome [55] support our findings. A similar observation was previously made by Tu et al. [56] for pectinase SX6 from *Penicillium oxalicum* with two separate catalytic domains for pectin methylesterase and polygalacturonase activities.

Figure 2a–c also demonstrate the enzyme-binding sites as predicted for cel12B, cel8C, and peh28 sequences, respectively, based on analysis using the 3D-LigandSite recognition server [31]. Twelve residues were identified in the predicted binding site for cel12B as shown in Fig. 2a. The two carboxylated glutamate residues, Glu^158^ and Glu^246^, are the catalytically active nucleophile and acid/base residues suggested for cel12B based on the similarity to those of Gloster et al. [52] for endo-β-1,4-glucanase B from *B. licheniformis*. Proline residue, Pro^170^, at the cel12B active site (Fig. 2a), may represent the C-terminus of an acidic flexible linker (FL) in the demonstrated structure. A similar residue was suggested before to form hydrogen bonds with the central protein region for a related GH-12 cellulase structure, LC-CelA, from *Rhodothermus marinus*, providing stability to the defined system [57]. The presence of an FL-related domain (Fibronectin type 3 domain, accession # cl21522 [58]) was found in the cel12B sequence using the NCBI-CD-BLAST server (results not shown). The FL has been reported to catalyze separation of a hydrophobic signal peptide that anchors the enzyme to the cell from the catalytic core [57, 59], and also to play a critical role in the enzyme processivity on crystalline cellulose [60, 61]. Future investigations of the predicted structure are planned to be carried out using site-directed mutagenesis and other related methodologies.

On the other hand, Glu^57^, Tyr^244^, Asp^245^, and Phe^335^ were the only residues identified for cel8C in the predicted binding site (Fig. 2b), which dominate the groove center of a substrate-binding pocket [62], (Fig. 2b). Glu^57^ and Asp^245^ are the catalytically active residues suggested for cel8C by comparison to that of Mazur and Zimmer [53].

Peh28 residues Ser^27^, Asp^28^, Ser^29^, Arg^30^, Asn^237^, Asn^265^, and Asn^290^ were identified in the active site of the predicted structure, as shown in Fig. 2c. These residues comprise the potential *N*- and/or *O*-glycosylation sites similar to those reported by van Pouderoyena et al. [54]. However, the similarity to *A. niger*’s endo-polygalacturonase I conserved domain sequences (Fig. 2d), suggests Asp^228^, Asp^249^, Asp^250^, and His^277^ to be the catalytic acid/base residues for peh28.

Other molecular target motifs were also noted for peh28 such as Cys^115^ and Trp^160^ at the peripheral loop region, and Asn^370^, Val^367^, Val^368^, Trp^351^, and Val^330^, were found proximal to the C-terminus in the enzyme model structure (Fig. 2c). Similar residues have been previously investigated for hydrophobicity and/or protein stability related functions in the protein structure of polygalacturonase PehA from *Erwinia carotovora* (*P. carotovorum*) [63]. The contribution of such identified residues in the peh28 stability could be validated in future studies through site-directed mutagenesis.

## Biochemical characterization of recombinant cel12B, cel8C, and peh28

### Purification of cel12B and cel8C and polygalacturonase

The crude protein extracts, partially purified with B-PER accessory reagents, were desalted and concentrated by multiple phases of ultrafiltration using PES membranes with different MWCs. The concentrated fractions were subjected to further purification by gel filtration using Sephadex G-100. A summary of each purification step is depicted in Table 2 for cel12B, cel8C, and peh28. The ultimate purification-fold values were calculated to be 17.4, 6.2, and 6.0 for cel12B, cel8C, and peh28, respectively. SDS-PAGE following final gel filtration revealed the appearance of other protein bands along with the identified enzyme bands as investigated before [8]. The presence of such impurities of other protein bands along with the enzyme bands suggests partial purification of the three enzymes. Similar observations have been previously reported by Tari et al. [64] for exo-polygalacturonase from *Aspergillus sojae*, who suggested that stability of the enzyme might be negatively affected by their purification due to the possible synergistic effect from other proteins found in solution with the desired enzyme components as originally proposed by Naidu and Panda [65]. Thus, the partially purified cel12B, cel8C, and peh28 are further characterized in the subsequent sections.

**Table 2:** Purification steps of cel12B, cel8C, and peh28 overexpressed in *E. coli* ^a^

Enzyme	Purification method	Fractions	Total enzyme activity (units)^c^	Total protein (mg)^d^	Specific activity (U/mg protein)	Purification fold	Yield (%)
cel12B	Crude extract^b^		4.49	73.2	0.061	0	100
	UF^e^-PES^f^—MWC^g^ (100 kDa)	Permeate	3.73	38.0	0.098	1.6	83.1
	UF-PES—MWC (50 kDa)	Permeate	2.76	11.0	0.251	4.1	61.4
	UF-PES—MWC (30 kDa)	Permeate	1.91	2.32	0.823	13.4	42.6
	Gel filtration^h^		1.32	1.24	1.06	17.4	29.3
cel8C	Crude extract		28.7	75.3	0.381	0	100
	UF-PES—MWC (100 kDa)	Permeate	25.8	44.5	0.579	1.5	89.7
	UF-PES—MWC (50 kDa)	Retentate	23.1	27.4	0.844	2.2	80.5
	UF-PES—MWC (30 kDa)	Permeate	18.2	7.39	2.47	6.5	63.4
	Gel filtration		14.4	6.08	2.36	6.2	50.0
peh28	Crude extract		1.58	88.8	0.0178	0	100
	UF-PES—MWC (100 kDa)	Permeate	1.37	52.2	0.0262	1.5	86.5
	UF-PES—MWC (50 kDa)	Retentate	1.31	31.2	0.0419	2.4	82.6
	UF-PES—MWC (30 kDa)	Permeate	1.00	11.3	0.0888	5.0	63.4
	Gel filtration		0.708	6.56	0.108	6.0	44.7

All values are given as a mean of triplicates ± SE

^a^Cel12B, cel8C, and peh28 are clones of *Pcc* for genes encoding cellulase B, cellulase C, and polygalacturonase, respectively, that were transformed into *E. coli* using pTAC-MAT expression vector

^b^Crude extracts are cell-free extracts of *E. coli* cell-free lysates. The cultures were stimulated for enzyme induction for 5 h, for cel12B and cel8C, and for 7 h, for peh28, at 37 °C using 10 mM IPTG

^c^One Unit of enzymatic activity is defined as the amount of enzyme releasing 1 µmol of reducing sugars per minute from the substrate under the assay conditions (pH 5.0 at 40 °C, for cel12B and cel8C, and pH 5.0 at 40 °C, for peh28)

^d^All protein concentrations are in mg per ml of enzyme solution at each fractionation stage

^e^Ultrafiltration

^f^Polyethersulfone

^g^Molecular weight cut-off

^h^Gel filtration was carried out using Sephadex G-100

### Mode of enzyme action and substrate specificity of cel12B and cel8C

Exoglucanase, endoglucanase, and β-glucosidase activities were determined for cel12B and cel8C using 20 mg/ml of Avicel, 20 mg/ml of CMC, and 10 mM of *p*-NPG, respectively. The soluble cellulose derivative, CMC, is commonly used as substrate for endolytic-cellulase activities [66], while Avicel is a crystalline cellulose preparation, similar in crystallinity index to pretreated natural cellulose [67]. Both amorphous and crystalline cellulosic regions are part of the natural cellulose framework but the latter regions contribute to the complexity of the material’s enzymatic degradation [67]. The data in Table 3 indicate that both cellulases showed an apparent endolytic activity on CMC. However, activity on Avicel was not detected with cel8C. Moreover, the activity determined for cel12B on CMC was minimal compared with those reported for several typical endoglucanases such as those of Irwin et al. [68]. The activity found for cel12B on Avicel as well as its minimal activity on CMC would suggest that it is an atypical endoglucanase. The corresponding Avicelase activity has been previously reported with related GH-12 cellulases such as those from *Trichoderma reesei* [69]. The presence of the CBD-II site noted above in the cel12B protein sequence would suggest an exolytic function on Avicel’s crystalline domains. An increase in the enzyme-binding affinity to the cellulose crystalline parts was previously reported in the presence of a CBD-II related structure [41]. The lack of the corresponding CBD motif in the cel8C protein sequence may explain its inactivity on Avicel. Mazur and Zimmer [53] also reported the absence of a CBD site in a related GH-8 cellulase. The high activity observed on CMC, as compared with cel12B, would suggest cel8C is a typical endoglucanase. The lower CMCase activity of cel12B might be related to the CBD and its inhibition of enzyme desorption following adsorption on the substrate [70, 71]. Table 3 shows that neither cel12B nor cel8C activities were detected on *p*-NPG and, thus, the enzymes’ β-glucosidase function can be dismissed. Similar findings have been reported for endoglucanases from different sources tested on *p*-NPG and cellobiose as substrates [72, 73].

**Table 3:** Exoglucanase, endoglucanase, and β-glucosidase activities of cel12B and cel8C on Avicel, CMC, and *p*-NPG, respectively

Enzyme	Exoglucanase activity (U/ml)^a^	Endoglucanase activity (U/ml)	β-Glucosidase activity (U/ml)
Cel12B	1.53 ± 0.09	1.21 ± 0.02	–
Cel8C	–	14.7 ± 0.6	–

The reactions were conducted for 1 h at 45 °C, pH 5.0 using 20 mg/ml Avicel or CMC for exoglucanase and endoglucanase activities, respectively, and for 30 min at 50 °C, pH 5.0 using 10 mM *p*-NPG for β-glucosidase activity

All values are given as a mean of triplicates ± SE

^a^A unit of enzyme activity (*U*) is defined as the amount of enzyme releasing 1 µmol of reducing sugars per minute for exoglucanase and endoglucanase activities on Avicel and CMC, respectively, and 1 µmol *p*-nitrophenol per minute for β-glucosidase activity on *p*-NPG under the assay conditions

### Kinetics of recombinant cel12B, cel8C, and peh28 with their respective substrates

Enzyme kinetic parameters, *V*_max_, *K*_m_, and *k*_cat_ and *k*_cat_/*K*_m_, given in Table 4, were estimated using direct fit to the Michaelis–Menten equation (figures not shown) over a 1–40 mg/ml range of CMC for cel8C and cel12B, and a 0.05–0.55 mg/ml range of polygalacturonic acid for peh28. All kinetic measurements were carried out at 45 °C for cel12B and cel8C, and 40 °C, for peh28 using the appropriate buffer system at pH 5.0. Cel12B exhibited 16-fold lower activity with CMC than cel8C as shown by the corresponding *V*_max_ values. However, the *K*_m_ value of cel8C with CMC, 35 mg/ml, was about twofold higher than that of cel12B on CMC but the *K*_m_ values of both enzymes on CMC are lower than those reported for other cellulases by Kim et al. [74] and Lin et al. [75] for modified endoglucanases of EngZ (K94R/S365P) and cel8 M at 42.5 °C, pH 7.0 and 40 °C, respectively. On the other hand, cel12B’s catalytic efficiency in terms of *k*_cat_/*K*_m_, 0.14 ml/mg/s, was 17-fold lower than that of cel8C on CMC (Table 5), which is eight times higher than that of a modified thermally stable endoglucanase EngZ (K94R/S365P) [74]. Although cel12B pales in comparison to cel8C, it displays a similar *k*_cat_ value, 2.7 s^−1^, to that reported by Okada et al. [19] for a related GH-12 endoglucanase from *T. reesei*. The difference in the catalytic performance of cel12B and cel8C on CMC might be attributed to the dissimilarities in their substrate preferences as well as the presence of a CBD as discussed above in the substrate specificity section. Preliminary kinetic assessment of cel12B acting on the solid substrate Avicel was also consistent with a Michaelis–Menten model (data not shown) but consideration of it will be left to a future study in order to incorporate mass-transfer effects to and from the solid surface into the model, as discussed by Cruys-Bagger et al. [76], and also to consider heterogeneity within Avicel itself between its crystalline and amorphous regions [77, 78].

**Table 4:** Enzyme kinetic parameters for cel12B, cel8C, and peh28^a^

Enzyme	Substrate	*V*_max_^b^ (µmol/ml/min)	*K*_m_^c^ (mg/ml)	*k*_cat_^d^ (s^−1^)	*k*_cat_/*K*_m_^e^ (ml/mg/s)
cel12B	CMC	2.4 ± 0.2	19 ± 3	2.7 ± 0.2	0.14 ± 0.03
cel8C	CMC	39 ± 4	35 ± 6	85 ± 9	2.5 ± 0.7
peh28	Polygalacturonic acid	2.0 ± 0.5	0.87 ± 0.29	4.2 ± 1.0	4.9 ± 2.8

^a^The parameters were determined at 40 °C and pH 5.0 for peh28 using 0.05–0.55 mg/ml polygalacturonic acid and at 45 °C and pH 5.0 for cel12B and cel8C using 1–40 mg/ml CMC. Parameters are given as a mean of triplicates ± SE

^b^Maximum velocity (at substrate saturation)

^c^Michaelis–Menten constant (half-saturation constant)

^d^Turnover number (enzyme concentration-independent specific rate at saturation)

^e^Catalytic efficiency (specificity constant)

**Table 5:** Fragmentation patterns of selective fragment ions (SFI) for trimethylsilyl and trimethylsilyl-oxime derivatives as analyzed by GC–MS^a^

Compounds	Trimethylsilyl and trimethylsilyl-oxime derivatives	Derivative structure	Molecular weight (g/mol)	Retention time (min)	Selective total fragment ions (SFI) *m*/*z* ^a^
Salicin internal standard (INSD)	Salicin-5-(TMS)		633.157	INSD:9.123	73, 147, 217, 361
Citric acid (CA)	Citric acid (4TMS)		480.848	CA:4.716	73, 147, 273
α- and β-d-glucopyranose (G 1-2)	d-Glucopyranose, 1,2,3,4,6-pentakis-O-(TMS)-		541.0615	G1:5.294 G2:5.859	73, 147, 191, 204
Glucose (*syn/anti*-oximes) (G 3-4)	Glucose-oxime-hexakis-(TMS)		628.2572	G3:6.042 G4:6.185	73, 147, 205, 319
α- and β-d-cellobiose (C 1-2)	d-glucopyranose, 4-*O*-[1,2,3,6-tetrakis-*O*-(trimethylsilyl)-β-d-glucopyranosyl]- -2,3,4, 6-tetrakis-*O*-(trimethylsilyl)-		919.7454	C1:10.700 C2:10.855	73, 147, 204, 361
α- and β-d-Galactopyranuronic acid (GA 1-2)	d-Galacturonic acid, *O*-pentakis (TMS)		555.0450	GA1:5.436 GA2:5.591	73, 147, 218
Galacturonic acid (*syn/anti*-oximes) (GA 3-4)	Galacturonic acid oxime-hexakis-*o*- (TMS)		618.22	GA3:6.328 GA4:6.539	73, 147, 218, 333
d-galactose (Gal)	Galactose oxime-hexakis (TMS)		628.2572	Gal:5.962	73, 147, 205, 319

The identified derivatives represent the mono- and di-saccharide products estimated throughout a 3-h period by cel12B, cel8C, and/or peh28 during the course of hydrolysis on their respective substrates

^a^The mass spectra and characterization of the derivatized compounds are shown in detail in Fig. 6 for INSD, CA, G1, G2, G3, G4, C1, and C2 and Fig. 7 for GA1, GA2, GA3, GA4, and Gal. The *m*/*z* represents the masses of the fragmentation ions detected for each theoretically derivatized compound relative to the corresponding abundance in integrator units/ng (Iu/ng) as shown in Figs. 6 and 7

Table 4 indicates that the *K*_m_ of peh28 with polygalacturonic acid, 0.87 mg/ml, is similar to those of commercial polygalacturonases [25]. On the other hand, the *V*_max_ of peh28 on polygalacturonic acid, 2.01 µmol/ml/min at 40 °C and pH 5.0, is higher than those of Ortega et al. [79], for commercial pectinases at 30 °C and pH 4.2, and lower than that of Joshi et al. [80], for a marine pectinase from *Bacillus subtilis* at 40 °C and pH 8.0. Such variations in *V*_max_ might be due to the dissimilar reaction conditions, including enzyme molar concentration; the better comparator would be *k*_cat_, if those concentrations were known. Activities of polygalacturonases are generally affected by the substrate-esterification, substrate surface charges, and the enzyme pI values [81]. Low substrate-esterification, for instance, can lower the enzymatic activity by increasing the non-productive binding as reported for polygalacturonic acid with an endo-polygalacturonase from *Verticillium alboatrum* [81]. Exo-polygalacturonases were also found to have lower activities than endo-polygalacturonases as reviewed by Niture [25]. Thus, the lower activity found for peh28 relative to those of Joshi et al. can be due to the low degree of esterification of polygalacturonic acid or due to the possible enzyme exolytic action on the substrate. The catalytic efficiency of peh28 was 4.87 ml/mg/s, which was higher than those reported by Maisuria et al. [82] at pH 8.5 and 50 °C and Joshi et al. [80] at pH 9.0 and 40 °C for polygalacturonases/pectinases from different sources. These observations indicate the industrial potential of peh28 and also highlight the importance of feedstock characterization for maximum biomass conversion by the tested enzyme.

### pH and temperature optima for substrate conversions with the recombinant enzymes

**Fig. 3:**
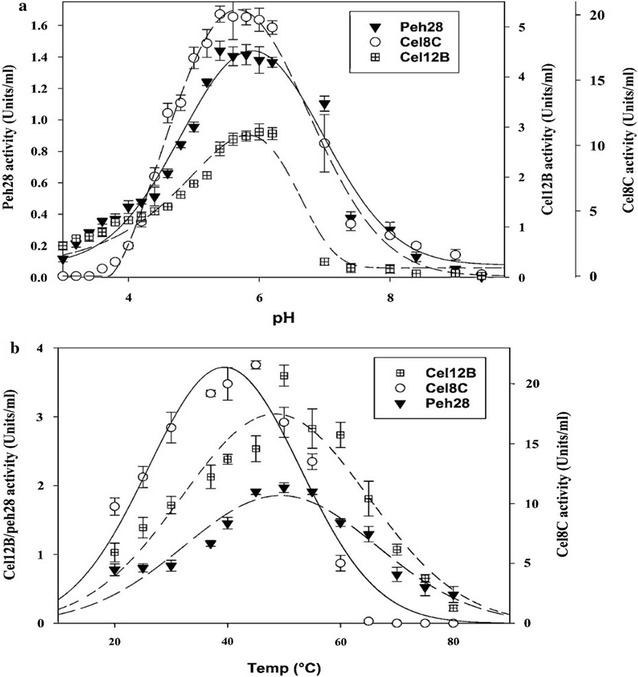
**a** pH profile of recombinant cel12B, cel8C, and peh28 incubated for 1 h at 40 °C with 25 mg/ml CMC, for cel12B and cel8C, and 4 mg/ml polygalacturonic acid, for peh28. **b** Temperature profile of recombinant cel12B, cel8C, and peh28 incubated for 1 h at pH 5.4 with 25 mg/ml CMC for cel12B and cel8C, and 4 mg/ml polygalacturonic acid for peh28. One unit of enzyme activity was defined as the amount of enzyme releasing 1 µmol of reducing sugars per minute from the substrate under the assay conditions. Values presented are given as a mean of triplicates ± SE

The optimum pH values for enzyme activities were investigated over a broad pH range of 3.0–10.0. Reactions were conducted for 1 h at 45 °C using 25 mg/ml of CMC for cel12B and cel8C, and at 40 °C using 4 mg/ml of polygalacturonic acid for peh28. The pH profile for the enzymes is shown in Fig. 3a. No activity was detected for cel8C at pH 3.0–3.4 but activity was detected at pH 3.6. On the other hand, cel12B and peh28 showed activity at all the tested pH levels. However, all enzymes displayed their maximum activities at a pH range of 5.4–6.2. Similar pH optima have been previously reported with other cellulases/endoglucanases of related GH-8 and GH-12 families and polygalacturonases/pectinases of related GH-28 family such as those reported by Yeh et al. [72] for a GH-12 endoglucanase from rice straw compost, Lin et al. [75] for a GH-8 cellulase from *E. coli* K12 strain, and Maisuria et al. [83] and Maller et al. [84] for GH-28 polygalacturonases from *Pcc*-BR1 and *Aspergillus niveus*, respectively. Figure 3a also displays a similar decline in activity for each enzyme in the alkaline pH range, 7.0–10. The enzymes exhibited variations in the decrease in activity at pH 7.0 as shown by the corresponding 90, 50, and 78% reductions from optimal activity for cel12B, cel8C, and peh28. Such significant decline in cel12B and cel8C activities at high pH has been seen before in related glycoside hydrolase families such as a GH-12 endoglucanase from *T. reesei*, a GH-12 xyloglucanase from *Fusarium graminearum* and a GH-8 cellulase mutant from *E. coli* K12 strain, as reported by Karlsson et al. [69], Habrylo et al. [85], and Lin et al. [75], respectively. Based on these findings, pH 5.4 was selected as optimal for all subsequent experiments.

Temperature optima for enzyme activities were determined over a range of 20–80 °C at pH 5.4, using 25 mg/ml of CMC for cel12B and cel8C, and 4.0 mg/ml of polygalacturonic acid for peh28. The temperature profiles for the enzymes shown in Fig. 3b indicate that the cel8C exhibited a different temperature-dependence than cel12B and peh28. In fact, there was complete inactivation of cel8C but not cel12B and peh28 at temperatures higher than 60 °C. The lower stability of cel8C has been previously noted for several cellulases of the GH-8 family [75]. Significant increases in enzyme activities were observed over the temperature range 20–45 °C, each reaching a maximum about 45 °C. Optima of 45 °C were previously found for several GH-8 cellulases as discussed by Lin et al. [75]. Activity of cel8C gradually decreased by 23–77% as the temperature increased from 50 to 60 °C relative to the activity at 45 °C (Fig. 3b). On the other hand, no significant change was observed in cel12B and peh28 activities when the temperature increased from 45 to 55 °C (Fig. 3b). Thus, cel12B and peh28 exhibited their maxima over a broad temperature range of 45–55 °C. Similar optimum temperatures have been previously reported with related GH-12 cellulases and GH-28 polygalacturonases from different sources as reported by Karlsson et al. [69], Amore et al. [86], and Picart et al. [87] for cellulases, and Kaur et al. [88] for polygalacturonase. The stability of cel12B and peh28 at high temperatures was shown by their 77, 52–66, and 30% activities retained at 60, 65, and 70 °C, respectively, relative to their average activities over 45–55 °C. The activity retained by peh28 at 60 °C or higher was atypical compared to other GH28-polygalacturonases such as that of NfPG I from Pan et al. [43]. The kinetic and thermodynamic stabilities of the current modified systems at industrially relevant temperatures will be discussed in detail in a future publication.

### Examination of synergism of cel12B and cel8C on Avicel and CMC substrates

**Fig. 4:**
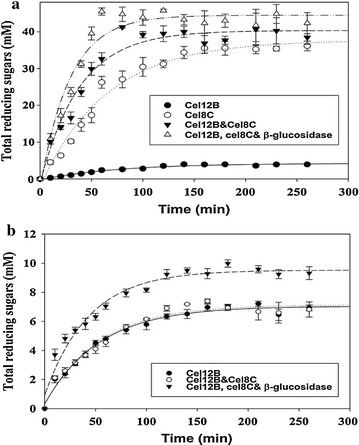
Test of synergism among recombinant products of cel12B and cel8C with/without β-glucosidase as compared to their individual actions on **a** CMC and **b** Avicel substrates. The reactions were conducted for 3 h with the activities being tested every 10 min in the first hour and every 20 min in the next 2 h at 45 °C using 2.5% (w/v) CMC/Avicel in 50 mM sodium citrate buffer (pH 5.4). Values presented are given as a mean of triplicates ± SE

An experiment was carried out to examine the potential synergy of cel12B and cel8C to achieve maximum hydrolysis on CMC and Avicel substrates. Total quantities of reducing sugars formed in separate and combined reactions of cel12B and cel8C with the substrates were measured and compared. The synergetic response was also investigated in the combined activities of cel12B and cel8C on each substrate in the presence of β-glucosidase. All synergies were investigated at intervals throughout a 3 h period of incubation at 45 °C and pH 5.4 using CMC and Avicel at 25 mg/ml as shown in Fig. 4a, for CMC, and 4b, for Avicel. Figure 4a shows that a cel12B and cel8C mixture achieved maximum substrate conversions at 80 min which was half the time for the individual enzymes on CMC. There was also a significant 1.4-fold increase in the corresponding total reducing sugars formed as compared with the sum of their individual activities on CMC. Synergism of endoglucanases on CMC has been reported by Rao et al. [89] and Zhou and Ingram [90] for endoglucanases from *Fusarium lini* and *Erwinia chrysanthemi*, respectively.

The synergism displayed by cel12B and cel8C on CMC may be correlated with their retention and inversion modes of action, respectively, on the substrate as investigated earlier [8]. This, in turn, might be due to the difficulty cel12B has to demonstrate its retention mechanism on cellohexaose-like substrates compared to smaller degradation products, e.g., cellotetraose and cellopentaose as explained below (see Product analysis section, below). Zhou and Ingram [90], explained the synergy between two endoglucanases, CelZ and CelY, from *E. chrysanthemi*, as due to the inability of CelY to utilize the soluble degradation products cellotetraose and cellopentaose which could be readily utilized by CelZ. Products averaging 10.7 glucosyl units were reported by the authors for the action of CelY, while average fractions of 3.6 glucosyl units arose by the combined action of CelZ and CelY. The lower activity found for cel12B by itself on CMC is similar to that of CelZ from *E. chrysanthemi*. Moreover, the CelY from *E. chrysanthemi* was assigned to the same GH-8 family ascribed to the present cel8C. These observations suggest relative substrate preferences as a possible mechanism for the enzyme synergy observed with cel12B and cel8C. Zhou and Ingram also reported that sequential hydrolysis of CMC by their two enzymes, CelZ and CelY, improved the synergy between them, when CelY was used first. They suggested that CelY increased the substrate digestibility for the ensuing action of CelZ on the partially hydrolyzed CMC. Thus, the low synergy observed herein might be improved if similar sequential hydrolysis had been used, cel8C first, then cel12B. Further investigation of the complementary actions of cel8C and cel12B is a promising focus for future research.

On the other hand, no detectable activity was shown on Avicel in the case of cel8C over all periods of incubation unlike cel12B (Fig. 4b). The maximum total reducing sugar products achieved on Avicel by cel12B was about 7.0 mM at 180 min incubation. No significant change was detected in the hydrolysis of Avicel when cel12B was combined with cel8C. This may be due to Avicel’s high level of crystallinity that prevents the enzyme access and, hence, the synergism as implied by Kostylev and Wilson [91]. Absence of a CBD in the cel8C sequence could explain the enzyme’s inactivity on crystalline cellulose as discussed above. The absence of cellulase synergy on crystalline cellulose has been reported with cellobiohydrolase I and endoglucanases I and II using cellulose microcrystals [92].

The insignificant activity observed for cel8C on Avicel and/or its lack of synergy with cel12B might be attributed to the formation of long insoluble products of six or more glucosyl units by cel8C that tend to not be further hydrolyzed and, in turn, must be removed by centrifugation prior to analysis. A similar explanation has been given for CelY and CelZ from *E. chrysanthemi*, for their lack of synergy on Avicel [90]. On the other hand, the synergy of CelY and CelZ on CMC was explained due to the formation of intermediate fragments by CelY that could be further utilized by CelZ to form more diffusible substrates and/or products [90], similar to the synergy observed for the present cel8C and cel12B on CMC.

Activity stimulation of 28–30% was observed when β-glucosidase was added to the mixture of cellulases using CMC or Avicel as substrates (Fig. 4a, b). Similar activity stimulation has been previously reported with β-glucosidase in conjugation with other cellulases from different sources such as those reported by Zhang and Lynd [93], Ng et al. [94], and Zhao et al. [95]. β-Glucosidase may enhance the cellulases’ function by eliminating the cellobiose-mediated inhibition encountered in many cellulolytic systems through the conversion of cellobiose to glucose as proposed by Andrić et al. [96], Ng et al. [94], and Zhao et al. [95]. The time for maximum total reducing sugar products from the mixture of cellulases on CMC was reduced to 60 min in the presence of β-glucosidase as shown in Fig. 4a. No change was detected, however, in the duration taken for cel12B to achieve maximum activity on Avicel upon β-glucosidase addition as shown in Fig. 4b. Those variations in the duration of incubation needed to achieve maximum total reducing sugar product formation on each substrate might be attributed to the formation of less hydrolyzable cellulose clusters generated as time progresses, as discussed by Turon et al. [97]. The synergy shown by the cellulases on Avicel or CMC may highlight the candidacy of such tailored catalyst cocktails for lignocellulosic biomass conversion. Further investigations using high-resolution microscopy are suggested for improved understanding of the mechanism of enzyme synergy for maximal biomass saccharification using the present enzymes. Adjusting the relative enzyme molar concentrations and understanding the kinetics of enzyme synergies are also anticipated milestones in our ongoing studies to achieve maximum enzymatic conversion of the substrates [98].

### Product analysis

**Fig. 5:**
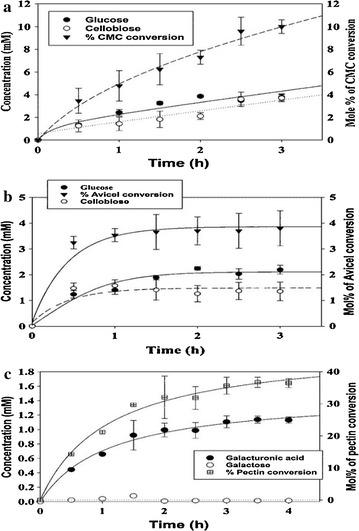
Product profiles and extents of polysaccharide conversion. **a** Glucose and cellobiose from CMC through combined activities of cel12B, cel8C, and β-glucosidase; **b** Glucose and cellobiose from Avicel through combined activities of cel12B, cel8C, and β-glucosidase; **c** Galacturonic acid (monogalacturonate) and galactose from pectin through activity of peh28. All reactions at 45 °C and pH 5.4; all sugar products were detected as trimethylsilyl and/or trimethylsilyl-oxime derivatives using GC–MS. Values presented are given as a mean of triplicates ± SE

**Fig. 6:**
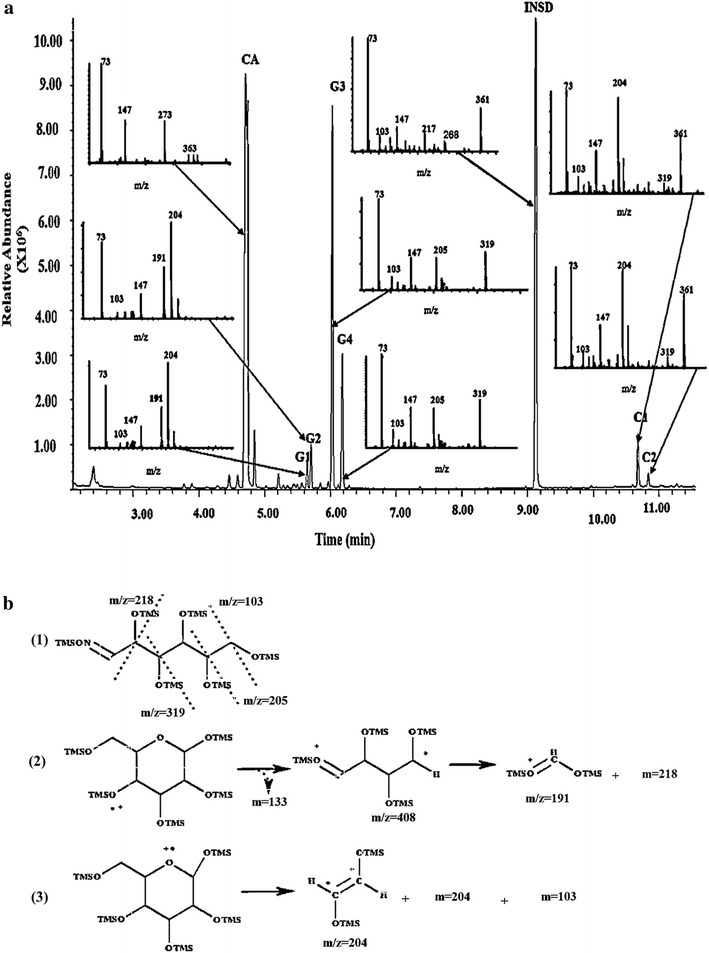
**a** GC–MS total ion chromatogram (TIC) and mass spectra (insets) of the trimethylsilyl (TMS) and trimethylsilyl-oxime (TMS-oxime) derivatives for compounds of CMC hydrolysis at 45 °C and pH 5.4 using a cel12B, cel8C and β-glucosidase cocktail **b** predicted fragmentation pattern showing the prominent mass ions of O-pentakis-TMS, (*1*), and oxime-hexakis-O-TMS, (*2*) and (*3*), derivatives of glucose relative to what was reported by Peterson [104] and Kennedy and Robertson [105], respectively. As shown in the elution profile, glucose (*G*) existing in two different configurations corresponds to that of the open-chain (oxime-hexakis-O-TMS), *G1* and *G2*, and cyclic-pyranose (O-pentakis-TMS), *G3* and *G4.* On the other hand, corresponding peaks for cellobiose are *C1* and *C2* which exist as the main product of CMC hydrolysis along with that of glucose. The two peaks of different retention time and similar fragmentation patterns detected for each of glucose and cellobiose derivatives represent the *alpha*- and *beta*-stereoisomers, in the case of TMS-glucose and cellobiose derivatives, and *syn*- and *anti*-oxime isomers in the case of TMS-oxime glucose derivative. The absence of those glucose and cellobiose peaks in GC-blank profile, figure not shown, confirms the current investigation. Other peaks such as *CA* and *INSD* were found to belong to citric acid buffer and salicin internal standard, respectively, according to mass spectrometric analysis

Investigation of cel8C and cel12B and β-glucosidase combined actions and the hydrolytic products formed over 3 h on Avicel or CMC has been carried out using gas chromatography coupled with mass spectrometry (GC–MS) as shown in Fig. 5a, b, respectively. Identification of each product generated during the enzymatic time courses on Avicel and CMC has been carried out using the retention times as well as the molecular ion fragments of their sugars given in Table 5 and Fig. 6.

Due to the GC limitations in quantification of the tri- and higher-oligomers, glucose and cellobiose were seen as dominant hydrolytic products from Avicel or CMC using the defined enzyme cocktail, as shown in Fig. 5a, b. In general, lower cellobiose and glucose concentrations arose from Avicel than CMC, which is likely a consequence of the soluble CMC being freely accessible whereas the solid Avicel is less accessible due to diffusional mass-transfer resistance. A similar explanation has been made for the GH-8, CelY, and GH-5, CelZ, endoglucanases from *E. chrysanthemi*, and their combined activities on Avicel and CMC [90]. This is further evidence of the inactivity of cel8C on Avicel, as discussed above.

Glucose and cellobiose were the hydrolysis products found in various combination reactions of other enzymes, such as CelY and CelZ [90], or individual actions of various GH-12 cellulases [69, 73, 98] on Avicel or CMC. This, in part, accounts for the progressive simultaneous activities of cel8C, as a typical endoglucanase, and cel12B, as non-typical endoglucanase, along with β-glucosidase on the substrates studied here. The variable substrate utilization by cel8C and cel12B was also considered with respect to their anomeric configuration-inverting and -retaining mechanisms, respectively, as reported earlier [8]. This may explain the partial dependency of cel12B on the preceding action of cel8C, to facilitate the retaining activity of the former through the actions on CMC. A similar explanation has been made previously for configuration-retaining cellulases [99], and for the synergy between CelZ and CelY endoglucanases [90]. Formation of cellotriose, with or prior to cellobiose, was also demonstrated with cellulases and/or endoglucanases having different modes of action as reported by Zhou and Ingram [90] and Karim et al. [99]. This supports the complementary roles suggested for cel12B and cel8C in their actions on CMC as explained above. Further investigation using isothermal calorimetry coupled with HPLC, could improve the understanding of end-product effects and/or enzyme synergy within the current modified system on each substrate.

Glucose concentration increased steadily in the CMC reaction over 2 h then leveled off (Fig. 5a). Cellobiose concentration, on the other hand, only varied after 1.5 h; the concentration at 2.0 h was almost double those at 1.5 h or earlier. The concomitant leveling off of glucose concentration after 2 h and the onset of cellobiose accumulation may be indicative of β-glucosidase inhibition by glucose, which has been seen before [95, 96]. This finding is in agreement with the maximum product formation shown at 80 min incubation as demonstrated above by the enzymes respective activities on CMC (Fig. 4a).

On the other hand, cellobiose and glucose concentrations were essentially constant over the time course for the enzyme cocktail acting on Avicel (Fig. 5b). This correlates with the enzymes’ inhibition at relatively lower concentrations of glucose and cellobiose, as compared to those from CMC. Variable sensitivity to end-product inhibition by both glucose and cellobiose was previously found among cellulases from similar sources and with different modes of action [100]. This suggests that continuous enzyme loading as the reaction proceeds might overcome the deactivation due to products formation. A similar suggestion has been made for analogous inhibition of GH-5 and GH-8 endoglucanases from *E. chrysanthemi* acting on Avicel [90].

The extent of substrate conversion was calculated for CMC and Avicel as 11.4 and 4.0%, respectively, based on the corresponding glucose and cellobiose products accumulated during the course of the cocktail activity on both substrates (Fig. 5a, b). It is also noted that the extent of substrate conversion did not vary over the course of enzymatic actions on Avicel, which is in agreement with the constant glucose and cellobiose concentrations observed over the course of reaction. Similar conversions to that achieved on Avicel have been previously reported for modified *Trichoderma* cellulase (Novozyme^®^ 50013) and β-glucosidase (Novozyme^®^ 50010) in their initial activities on Avicel which was attributed to the utilization of the easily accessible amorphous cellulose on the substrate surface [78]. However, these authors (Gao et al. [78]) observed an increase in the substrate conversion after prolonged incubation with Avicel, which is not the case with the current modified system. Gao et al. correlated the increase in the substrate conversion at prolonged incubation with consumption of the crystalline cellulose parts. Thus, the constant product concentrations and substrate conversion over the time course seen in the current study suggest that the enzymes were only capable of digesting the accessible amorphous substrate surfaces of Avicel, which they did rather quickly (first 30 min). Further analysis at longer incubation times may be required to examine the enzyme long-term stability and/or activity on crystalline and amorphous cellulose surfaces.

**Fig. 7:**
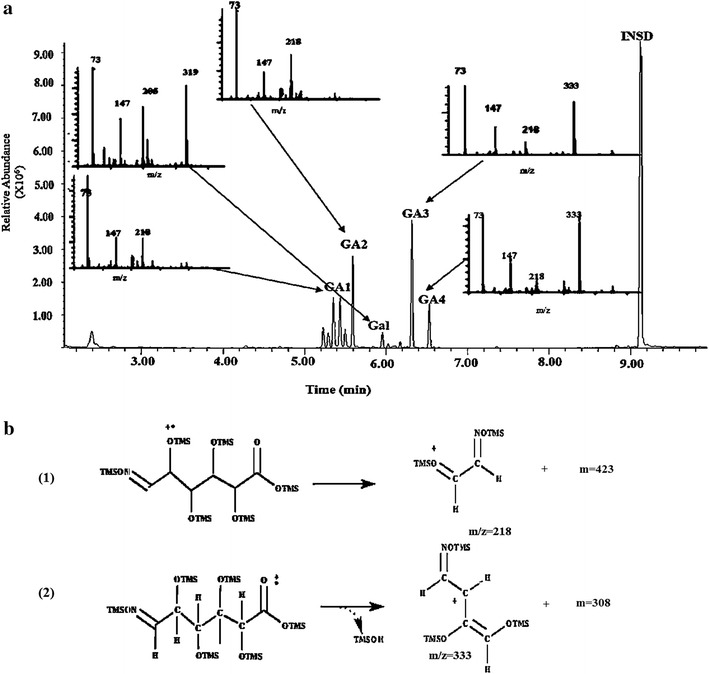
**a** GC–MS total ion chromatogram (TIC) and mass spectra (insets) of the trimethylsilyl (TMS) and trimethylsilyl-oxime (TMS-oxime) derivatives for compounds of pectin hydrolysis at 45 °C and pH 5.4 using peh28. **b** Predicted fragmentation pattern of oxime-TMS derivative of galacturonic acid showing prominent ions of *m*/*z* = 218, (**1**), and *m*/*z* = 333, (**2**), relative to what was reported by Peterson [104]. As shown in the elution profile, galacturonic acid (**GA**) exists in two different configuration forms of open-chain and cyclic-pyranose which correspond to TMS (**GA1-2**) and TMS-oxime (**GA3-4**) derivatives. Galacturonic acid is the main product displayed relative to the other less dominant compound galactose (*Gal*). Those components were not identified in the blank profile, figure not shown, and were expected as a result of pectin hydrolysis using peh28. Two peaks with different retention times and identical mass fragments were detected in case of TMS and TMS-oxime derivatives of galacturonic acid in their *alpha*- and *beta*-stereoisomers and *syn*- and *anti*-oxime stereoisomers, respectively

Peh28 activity on pectin over 4 h was also investigated by GC–MS, and the hydrolysis product concentrations are given in Fig. 5c. Identification of the peh28 hydrolytic products has been carried out using the corresponding retention time and molecular ion masses given in Table 5 and Fig. 7. Monogalacturonate was the main product detected for the peh28 activity on pectin over the time course as shown in Fig. 7. Galactose was also found as shown in Fig. 7, along with other minor peaks, which might be xylose, arabinose, and/or rhamnose as reported with other polygalacturonases from different sources [101, 102]. Figure 5c indicates that monogalacturonate concentration steadily increased until 1.5 h, then leveled off. On the other hand, galactose concentrations were much smaller and varied over the entire course of reaction (Fig. 5c).

Formation of monogalacturonate as a major hydrolytic product has been previously reported by Kuivanen et al. [101] and Mertens and Bowman [103], for polygalacturonases from filamentous fungi (*T. reesei * Δ*lgd1*and *A. niger* Δ*gaaB*) and *Rhizopus oryzae* RPG1, respectively. Formation of monogalacturonate products during the enzymatic reaction on pectin might be correlated with its processive action while continuously bound to the substrate, as explained by Mertens and Bowman [103]. This supports the processive function hypothesized for Arg^96^ in the corresponding peh28 model structure, similar to what was previously described by van Pouderoyen et al. [54] (See Sequence analysis and homology modeling section, above). Mertens and Bowman [103] also correlated monogalacturonate production with simultaneous enzyme action on multiple subsite loci in the binding to the substrate. This supports the anomeric configuration-retaining mode of action suggested for peh28 as previously explained [8]. Moreover, the monogalacturonate production along with the low specific activity demonstrated on polygalacturonate (Table 4) would support the enzyme’s dominant exolytic action on the substrate.

The extent of substrate conversion was calculated for peh28 in its activity on polygalacturonate as 36.5% over the 4 h period shown in Fig. 5c, based on the corresponding galacturonyl residues produced. This finding is not in agreement with that of Mertens and Bowman [103], who noted lower monogalacturonate production only in the first few minutes of the reaction, which may highlight the enhanced processivity and/or tolerance to end-product inhibition of the present peh28. The lower rate of conversion observed as the time progresses may, however, indicate substrate depletion and/or enzyme deactivation. Further investigation is thus necessitated to characterize enzyme behavior over a prolonged incubation period.

Production of glucose and cellobiose by the current-modified cellulases and β-glucosidase activities on Avicel or CMC as well as the monogalacturonate production by peh28 validates their relevance for industrial biofuel production. The process of product optimization over a prolonged time period will be necessary for achieving maximum hydrolysis using the current-modified enzymes system.

## Conclusions

The study provided some molecular characterization as well as biochemical analysis for the behavior of recombinant cel12B, cel8C, and peh28 enzymes, alone and in certain combinations, from *Pectobacterium carotovorum* subsp. *carotovorum* (*Pcc*) on cellulose and pectin substrates. The enzymes were assigned for their molecular similarity to glycoside hydrolase families 12, 8, and 28, respectively, and their catalytic domain residues were identified based on the analysis of their model structures. These outcomes suggest that some residues of cel12B and peh28 related to conformational and thermal stability are targets for further analysis. The presence of a CBD-II site in the cel12B sequence could partially explain the enzyme’s apparent function on the crystalline cellulose domains of Avicel. The relative thermal instability of cel8C at higher temperatures could also be predicted from its structural similarity to other GH-8 cellulases. The high catalytic activity of cel8C on CMC and the absence of similar activity on Avicel are correlated with typical endoglucanase characteristics. On the other hand, the lower CMCase activity of cel12B and its apparent activity on Avicel indicate atypical endoglucanase behavior. Enzyme processivity was concluded for peh28 from its close similarity to endo-polygalacturonase I from *A. niger* and from the existence of monogalacturonate as its dominant hydrolytic end-product. Moreover, the close sequence similarity to that of endo-polygalacturonase-I and pectate lyase-6 domains suggests the multi-domain activities of peh28. The relative substrate conversion values in terms of glucose and cellobiose formation from CMC and Avicel, and monogalacturonate from pectin for the current-modified system, suggest the enzymes’ candidacy for biofuel production. Site-directed mutagenesis, to promote sequential cel12B and cel8C hydrolysis and integration with other cellulolytic systems, is suggested for improving the cellulolytic synergy of the applied mixture. Overall, this study provides justification for further optimization of the enzymes’ catalytic performance in saccharification of lignocellulosic materials in future work.

## Abbreviations

cel12B: cellulase-12B
cel8C: cellulase-8C
peh28: polygalacturonase-28
GH: glycoside hydrolase
*Pcc*: *Pectobacterium carotovorum* subsp. *carotovorum*
*E. coli*: *Escherichia coli*
GH: glycoside hydrolase
GH-8: glycoside hydrolase family 8
GH-12: glycoside hydrolase family 12
GH-28: glycoside hydrolase family 28
CBD: carbohydrate binding domain
CMC: carboxymethyl cellulose
*p*NPG: *p*-nitrophenyl-β-d-glucopyranoside
CAZymes: Carbohydrate-Active enZymes
NCBI: National Center for Biotechnology Information
LB: Luria–Bertani
CDD: Conserved Domain Database
IPTG: isopropyl β-d-1-thiogalactopyranoside
DNS: dinitrosalicylic acid
NS: Nelson–Somogyi
SDS-PAGE: sodium dodecyl sulfate-polyacrylamide gel electrophoresis
RPS: reverse-position-specific
*K*_m_: Michaelis–Menten constant
*V*_max_: enzyme maximum velocity
*k*_cat_: enzyme turnover number
*k*_cat_/*K*_m_: enzyme specificity constant
SDS: sodium dodecyl sulfate
DTT: dithiothreitol
GC: gas chromatography
MS: mass spectrometry
MSD: mass selective detector
TIC: chromatograms from total ion current
SFI: spectra from selective fragment ion analysis
UF: ultrafiltration
PES: polyethersulfone
MWC: molecular weight cut-off
